# Economic value of extended-window intravenous alteplase in posterior circulation stroke: a Markov analysis based on the EXPECTS trial

**DOI:** 10.3389/fphar.2025.1734165

**Published:** 2026-01-08

**Authors:** Yudong Ma, Yake Lou, Maolin Chen, Yuhong Zeng

**Affiliations:** 1 Department of Neurosurgery, Air Force Medical Center, PLA, Beijing, China; 2 Interventional Center of Valvular Heart Disease, Beijing Anzhen Hospital, Capital Medical University, Beijing, China; 3 Emergency Medicine Clinical Research Center, Beijing Chaoyang Hospital, Capital Medical University, Beijing, China; 4 Clinical Research Center, Sichuan Provincial People’s Hospital, University of Electronic Science and Technology of China, Chengdu, Sichuan, China

**Keywords:** alteplase, cost-effectiveness, EXPECTS, Markov, stroke

## Abstract

**Objective:**

To evaluate the cost-effectiveness of intravenous alteplase compared with standard medical therapy alone for patients with posterior circulation ischemic stroke (PCIS) presenting 4.5–24 h after symptom onset, from the perspective of the Chinese healthcare system.

**Methods:**

A hybrid decision tree and Markov model was developed to simulate disease progression over a lifetime horizon (30 years). Clinical efficacy, utility values, and cost data were derived from the EXPECTS randomized controlled trial and domestic database. The primary outcome was the incremental cost-effectiveness ratio (ICER), expressed as cost per quality-adjusted life year (QALY) gained. Cost-effectiveness was assessed against a willingness-to-pay (WTP) threshold of one time China’s *per capita* GDP (95,749 CNY). One-way and probabilistic sensitivity analyses were performed to test robustness of results.

**Results:**

Compared with standard therapy, alteplase provided an additional 0.95 QALYs at an incremental cost of 17,580 CNY, resulting in an ICER of 18,567 CNY per QALY gained. This ICER is substantially below the WTP threshold, indicating that alteplase is highly cost-effective. The one-way sensitivity analysis showed that the ICER was most sensitive to the risk ratios of disability outcomes (mRS 2–5), yet remained within the cost-effective range across all parameter variations. The probabilistic sensitivity analysis confirmed that at the WTP threshold of 35,000 CNY per QALY, the probability of alteplase being cost-effective was nearly 100%.

**Conclusion:**

For patients with PCIS in the 4.5–24-h time window, intravenous alteplase is a highly cost-effective treatment option in China. This finding supports the broader implementation of extended-window thrombolysis for eligible PCIS patients, particularly in settings where access to endovascular thrombectomy or advanced perfusion imaging is limited.

## Introduction

Stroke is the third leading cause of death and disability, affecting an estimated 101 million people globally in 2019 (GBD, 2019 Stroke Collaborators, 2021). In China, there were 28.76 million stroke cases in 2019, about 84% of which were ischemic stroke, causing 2.19 million deaths and 45.9 million disability-adjusted life years (DALYs) (Ma et al., 2021). A central goal in the acute treatment of ischemic stroke is to restore cerebral perfusion as quickly as possible through intravenous thrombolysis (IVT), endovascular thrombectomy, or both (Hilkens et al., 2024). Alteplase, a tissue plasminogen activator (tPA), is the guideline-recommended IVT agent for eligible acute ischemic stroke (AIS) patients within 4.5 h after the onset of stroke (Berge et al., 2021; Powers et al., 2019). However, in clinical practice, most AIS patients fail to receive thrombolytic therapy within the optimal time window, a trend particularly pronounced in China (Tong et al., 2012; Yuan et al., 2022). In addition, potentially viable brain tissue may still exist 4.5 h after the onset of stroke, allowing patients to benefit from thrombolytic therapy. Therefore, exploring whether thrombolytic therapy beyond the optimal time window benefits selected patients with AIS has become an important research hotspot.

The Extending the Time for Thrombolysis in Emergency Neurological Deficits (EXTEND) trial was the first to extend the IVT time window of alteplase to 9 h in AIS patients with salvageable brain tissue and found a higher percentage of patients with a modified Rankin scale (mRS) score of 0 or 1 in alteplase group than that in the placebo group (35.4% vs. 29.5%, adjusted risk ratio [RR], 1.44; 95% confidence interval [CI], 1.01 to 2.06; P = 0.04) (Ma et al., 2019). Subsequently, the Tenecteplase Reperfusion Therapy in Acute ischemic Cerebrovascular Events-III (TRACE-III) trial demonstrated that administration of tenecteplase (a modified form of tPA) within 4.5–24 h in patients with anterior circulation large-vessel occlusion ineligible for endovascular thrombectomy were beneficial, which provides a new late time window treatment plan for patients with anterior circulation large vessel occlusion (Xiong et al., 2024). Tenecteplase is a genetically modified variant of alteplase with enhanced fibrin specificity and a longer half-life, and has been investigated as an alternative thrombolytic agent in extended time windows. More recently, the Extending the Time Window for Thrombolysis in Posterior Circulation Stroke without Early CT Signs (EXPECTS) trial showed that IVT with alteplase administered between 4.5 and 24 h significantly improved functional outcomes at 90 days compared to standard treatment in posterior circulation AIS patients without thrombectomy (mRS 0–2: 89.6% vs. 72.6%, adjusted RR, 1.16; 95% CI, 1.03–1.30; P = 0.01) (Yan et al., 2025). This is the first study to extend the IVT time window to 24 h in patients with posterior circulation AIS. However, in real-world clinical practice, cost-effectiveness represents a critical consideration alongside therapeutic efficacy when evaluating widespread implementation of extended-window thrombolysis. At present, one study had demonstrated that tenecteplase between 4.5 and 24 h is highly cost-effective for AIS patients without thrombectomy (Chen et al., 2025). As alteplase is more expensive than tenecteplase, whether IVT with alteplase within 24 h in posterior circulation stroke patients is also cost-effective needs further explore. This study aims to assess the cost-utility of alteplase therapy within this patient population from the perspective of healthcare system in China.

## Methods

This study was conducted and reported in accordance with the Consolidated Health Economic Evaluation Reporting Standards 2022 (CHEERS 2022) statement (Husereau et al., 2022). All data were obtained from published studies or publicly accessible databases. No individual patient information or data directly collected from patients were used; therefore, ethical approval was not required.

### Patients

This analysis modeled a cohort of patients with posterior circulation AIS, reflecting the population enrolled in the EXPECTS trial (Yan et al., 2025). Participants were adults who arrived 4.5–24 h after symptom onset, including those with wake-up or unwitnessed strokes where the estimated onset time met this window. Neurological impairment was confirmed by an NIHSS score of at least 1, and brain imaging demonstrated a PC-ASPECTS of 7 or higher on CT or MRI, indicating limited early ischemic change. Diagnosis was supported by both imaging findings and clinical presentation. All had a pre-stroke mRS below 2 and were not candidates for endovascular intervention. Individuals were excluded for factors such as contraindications to alteplase, substantial hemorrhage risk, recent major surgery or trauma, infarction in the anterior circulation, pregnancy, or any condition likely to compromise treatment safety, protocol adherence, or follow-up.

### Intervention

In the alteplase group, patients received intravenous alteplase at 0.9 mg per kilogram of body weight, not exceeding a total of 90 mg. The initial 10% of the dose was administered as a rapid bolus over 1 min, followed by infusion of the remaining 90% over 60 min. The standard treatment group was managed with antiplatelet therapy—such as aspirin, clopidogrel, or their combination—and other supportive measures, in line with the Chinese Guidelines for Diagnosis and Treatment of Acute Ischemic Stroke 2018 (Neurology and Society, 2018).

### Model structure

We developed a hybrid model combining a decision tree and a Markov model to evaluate the cost-effectiveness of alteplase plus standard medical therapy versus standard therapy alone in patients with posterior circulation ischemic stroke (PCIS) at 4.5–24 h after onset in China. The target population excluded patients with extensive early hypodensity on computed tomography and those scheduled for thrombectomy. The model simulated outcomes over a lifetime horizon of 30 years. This corresponds to a mean starting age of 64 years and an ending age of 94 years, which exceeds China’s average life expectancy of 79 years in 2024. Both the decision tree and the Markov cycles were set at 3-month intervals, resulting in a total of 119 Markov cycles. To minimize potential overestimation of costs and effects, a half-cycle correction was applied. From the perspective of the Chinese healthcare system, only direct medical costs were included; direct non-medical and indirect costs were excluded from the analysis. All future costs and health outcomes were discounted at an annual rate of 5%, in accordance with the China Guidelines for Pharmacoeconomic Evaluations (Guoen et al., 2020). For sensitivity analyses, a range of 0%–8% was applied to reflect plausible variations in long-term economic conditions.

**FIGURE 1:**
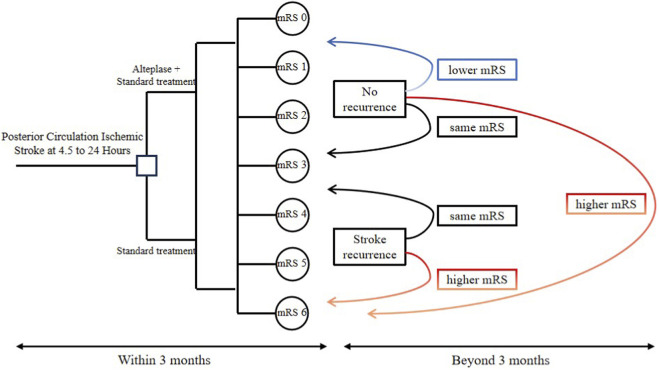
Schematic diagram of the decision tree and Markov model. In the decision tree model, patients with posterior circulation ischemic stroke could receive either standard treatment or standard treatment plus intravenous alteplase, resulting in different costs and effectiveness outcomes. The right section illustrates the long-term Markov model used to simulate lifetime costs and effectiveness. The incremental cost-effectiveness ratio was calculated based on total accumulated costs and effectiveness. mRS, modified Rankin Scale.

In the decision tree model (Figure 1), patients with posterior circulation stroke were randomly assigned to two groups: one receiving alteplase plus standard therapy, and the other receiving standard therapy alone. At 3 months, clinical outcomes, costs, and adverse events were assessed. The outcome measure of interest was the mRS, which reflects the level of disability, where mRS 0 indicates no disability, mRS 5 indicates the most severe disability, and mRS 6 represents death. Costs collected at 3 months included stroke hospitalization costs, post-discharge care costs, alteplase costs, and costs related to adverse events. The major adverse event considered was symptomatic intracranial hemorrhage (sICH), with its incidence derived directly from the EXPECTS trial (Yan et al., 2025).

After the initial 3-month decision tree phase, patients entered the Markov model, which was designed to capture long-term costs and outcomes after stroke. Since patients in the alteplase plus standard therapy group achieved better mRS scores at 3 months, their initial state distribution in the Markov model was more favorable than that of the standard therapy group. Each Markov cycle represented a 3-month period, with a total of 119 cycles over the lifetime horizon. In each cycle, patients could experience one of three events: no event, recurrent stroke, or death. Deaths were classified as either occurring subsequent to a recurrent stroke or resulting from background mortality. Patients who experienced no event remained in the same mRS state in the next cycle. Those with a recurrent stroke could remain in the same or move to a worse mRS state, while patients who died entered the absorbing state “Dead” and exited the simulation. The overall model structure is shown in Figure 1.

### Transition probability

In the decision tree model, the 3-month distribution of mRS outcomes was directly derived from the EXPECTS trial (Yan et al., 2025). In that trial, the proportions of patients in the alteplase group with mRS scores of 0–6 at 3 months were 0.3913, 0.3478, 0.1565, 0.0261, 0.0174, 0.0087, and 0.0522, respectively. The corresponding proportions in the standard therapy group were 0.2564, 0.3504, 0.1197, 0.1197, 0.0342, 0.0342, and 0.0855. The incidence of sICH was 0.0172 in the alteplase group and 0.0087 in the control group.

The initial mRS distribution in the Markov model was derived from the observed outcomes reported above. For subsequent cycle transitions, the EXPECTS trial did not provide annual recurrent stroke probabilities applicable to the Chinese population; therefore, recurrence rates were sourced from the IRIS trial, which demonstrated that higher mRS scores are associated with increased recurrence risk (de Havenon et al., 2024). The case-fatality rate after recurrent stroke was set at 0.21, derived from a separate study of Chinese stroke patients (Pan et al., 2018). Background mortality rates in the Markov model were sourced from the China Health Statistical Yearbook 2023 (Shili et al., 2024). Importantly, these rates were age-specific, ensuring that mortality risk increased appropriately with age throughout the model’s 30-year horizon. Because post-stroke patients experience higher mortality than the general population, the age-specific background mortality rates were further adjusted using a hazard ratio derived from prior studies to reflect excess post-stroke mortality (Chen et al., 2025; Chen et al., 2023).

### Cost

Only direct medical costs were included in this study. In this study, indirect costs were not included. Indirect costs typically refer to productivity losses resulting from morbidity or premature mortality, as well as the economic value of informal caregiving provided by family members. These costs reflect the broader societal burden of stroke but were excluded because the analysis was conducted from the perspective of the Chinese healthcare system, which considers only direct medical expenditures. All costs were converted to 2024 Chinese Yuan (CNY) values. Costs prior to 2024 were inflated to 2024 levels using the healthcare component of the Chinese CPI, while future costs were discounted at an annual rate of 5%.

For both the alteplase group and the standard therapy group, cost components included stroke hospitalization costs, post-stroke care costs, recurrent stroke costs, and adverse event costs. In addition, the alteplase group incurred the drug acquisition cost of alteplase. The hospitalization and post-stroke care costs varied according to mRS levels, with higher disability corresponding to greater resource utilization. These data were obtained from published studies on Chinese stroke patients. The cost of recurrent stroke was sourced from institutional data in China (Chen et al., 2023). The cost of sICH was derived from the Thrombolysis Implementation and Monitor of Acute Ischaemic Stroke in China (TIMS-CHINA) study, a national prospective registry including 1,440 AIS patients treated with intravenous tPA across 67 centers in China (Pan et al., 2018). The cost of alteplase was based on the national procurement price under China’s volume-based purchasing program, which is uniform nationwide: 1,700 CNY for 20 mg and 3,400 CNY for 50 mg. In the base-case analysis, the mean value of these two unit prices were applied, while in the one-way sensitivity analysis, the lower and upper bounds were defined by the respective prices of 50 mg and 20 mg formulations.

### Utility

Utility reflects the quality of life of patients, and health outcomes were expressed as quality-adjusted life years (QALYs), calculated as utility multiplied by life years. In our cost-effectiveness analysis, the utility values for each mRS state were obtained from a randomized controlled trial conducted in China (Wang et al., 2014). The utility scores for mRS levels 0 to 6 were 0.95, 0.89, 0.67, 0.44, 0.16, 0.10, and 0, respectively. For recurrent stroke, a utility value of 0.42 was applied, while for sICH, a disutility of 0.38 was incorporated into the model (Wang et al., 2024).

### Outcome

The primary outcome of this study was the incremental cost-effectiveness ratio (ICER), defined as the additional cost (in CNY) required to gain one additional quality-adjusted life year (QALY). According to the China Guidelines for Pharmacoeconomic Evaluations (Guoen et al., 2020), alteplase was considered not cost-effective if the ICER exceeded three times the *per capita* gross domestic product (GDP). When the ICER was between one and three times the *per capita* GDP, alteplase was deemed cost-effective, and when the ICER was below one time the *per capita* GDP, alteplase was regarded as highly cost-effective. Secondary outcomes included incremental cost, incremental effectiveness, incremental life years, total cost, total effectiveness, and total life years.

### Sensitivity analysis

Sensitivity analyses included both one-way sensitivity analysis and probabilistic sensitivity analysis (PSA). In the one-way sensitivity analysis, individual input parameters were varied within their respective confidence intervals to examine the impact of each variable on the ICER. The resulting changes in ICER values were illustrated using a tornado diagram, which highlights the relative influence of each parameter on model outcomes. The PSA accounted for the joint uncertainty across multiple parameters simultaneously. In the PSA, input variables were assigned appropriate probability distributions based on their characteristics: transition probabilities and utility values followed beta distributions, while cost parameters followed gamma distributions. The PSA was conducted to evaluate how combined parameter uncertainty influenced the ICER. Results were presented using scatter plots on the cost-effectiveness plane and cost-effectiveness acceptability curve.

## Results

### Base case analysis

Over a lifetime horizon, patients receiving standard treatment achieved 5.55 QALYs and 8.39 life years, with a lifetime cost of 127,227 CNY. In comparison, those treated with alteplase plus standard treatment achieved 6.49 QALYs and 8.90 life years, incurring a lifetime cost of 144,804 CNY. Thus, alteplase plus standard treatment provided an additional 0.95 QALYs (0.51 life years) at an incremental cost of 17,580 CNY, resulting in an ICER of 18,567 CNY per QALY gained, which is below one times China’s *per capita* GDP in 2024 (Table 1).

**TABLE 1:** Other clinical and cost input parameters and data sources.

Parameters	Value	Range	Distribution	Source
Outcomes at month 3 for patients in standard treatment				
mRS 0	0.2564	0.1773–0.3355	—	Yan et al. (2025)
mRS 1	0.3504	0.2640–0.4369		
mRS 2	0.1197	0.0609–0.1785		
mRS 3	0.1197	0.0609–0.1785		
mRS 4	0.0342	0.0013–0.0671		
mRS 5	0.0342	0.0013–0.0671		
mRS 6	0.0855	0.0348–0.1361		
Outcomes at month 3 for patients in alteplase				
mRS 0	0.3913	0.3021–0.4805	—	Yan et al. (2025)
mRS 1	0.3478	0.2608–0.4349		
mRS 2	0.1565	0.0901–0.2229		
mRS 3	0.0261	0–0.0552		
mRS 4	0.0174	0–0.0413		
mRS 5	0.0087	0–0.0257		
mRS 6	0.0522	0.0115–0.0928		
sICH incidence in standard treatment	0.0087	0–0.0409	β	Yan et al. (2025)
sICH incidence in alteplase	0.0172	0–0.0257	β	Yan et al. (2025)
Death hazard ratios				
mRS 0	1	1–1.2	—	Chen et al. (2023)
mRS 1	1	1–1.2		
mRS 2	1.11	0.89–1.3		
mRS 3	1.27	1.02–1.52		
mRS 4	1.71	1.37–2.05		
mRS 5	2.37	1.90–2.84		
Annual background mortality				
60-	0.00760	​	—	Shili et al. (2024)
65-	0.01266			
70-	0.02159			
75-	0.03731			
80-	0.06340			
85-	0.15120			

mRS, modified Rankin Scale; sICH, symptomatic intracranial hemorrhage.

### One-way sensitivity analysis

**FIGURE 2:**
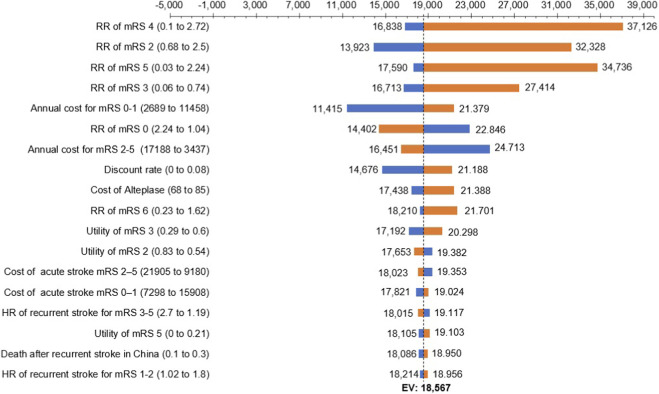
Tornado diagram of ICER across input parameters. Risk ratios for disability outcomes (mRS 2–5) exerted the greatest influence on ICER variation, though all results remained within the cost-effective range across parameter fluctuations. ICER, incremental cost-effectiveness ratio; RR, risk ratio; mRS, modified Rankin Scale; HR, hazards ratio.

As shown in Figure 2, the input parameters that had the greatest impact on the ICER were the RRs for mRS 4, mRS 5, mRS 2, and mRS 3. Other parameters—such as the proportion of patients with mRS 3 in the control group at 3 months post-stroke, the annual cost for mRS 0–1, the proportion of patients with mRS 5 in the control group at 3 months post-stroke, and the annual cost for mRS 2–5—also influenced the ICER, but to a lesser extent. Notably, variations in all parameters kept the ICER within the range of 11,000–38,000 CNY per QALY.

### PSA results

**FIGURE 3:**
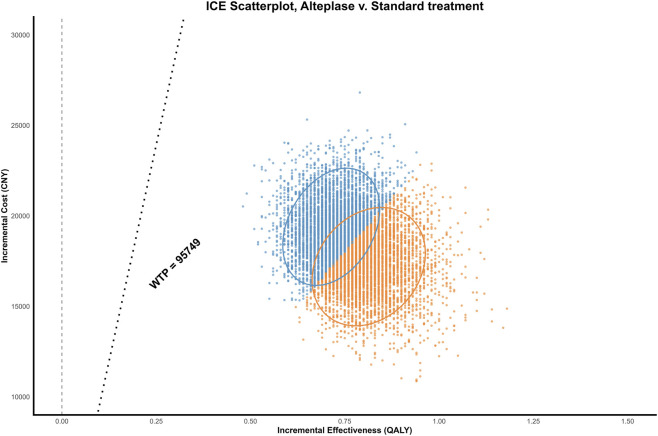
Incremental cost-effectiveness scatterplot. All scatter points lie in the first quadrant and below the willingness-to-pay (WTP) threshold of 95,749 CNY per quality-adjusted life year (QALY), indicating cost-effectiveness of alteplase compared with standard treatment. CNY, Chinese Yuan; ICE, incremental cost effectiveness.

**FIGURE 4:**
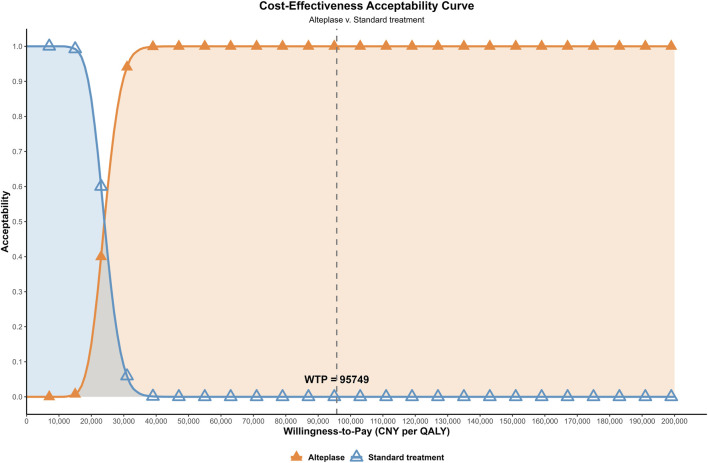
Cost-effectiveness acceptability curve. At a WTP threshold of 24,000 CNY per QALY, alteplase and standard treatment exhibited similar acceptability. When the threshold exceeded 35,000 CNY per QALY, the probability that alteplase was cost-effective approached 100%. WTP, willingness-to-pay; CNY, Chinese Yuan; QALY, quality-adjusted life year.

As shown in Figure 3, the incremental cost-effectiveness scatterplot indicated that all simulated points were located in the first quadrant and lie below the willingness-to-pay threshold line of 95,749 CNY per QALY. Figure 4 showed that when the willingness-to-pay was 24,000 CNY per QALY, alteplase and standard treatment had equal probabilities of being cost-effective. However, when the willingness-to-pay exceeded 24,000 CNY per QALY, the probability that alteplase was cost-effective increased, reaching nearly 100% when the willingness-to-pay reached 35,000 CNY per QALY.

## Discussion

This study is the first cost-effectiveness analysis of alteplase for PCIS within an extended 24-h time window. While the EXPECTS trial established the clinical efficacy of this regimen (Yan et al., 2025), its widespread adoption in real-world practice depends heavily on economic feasibility. Our findings demonstrate that IVT with alteplase for PCIS between 4.5 and 24 h is highly cost-effective compared with standard medical therapy in the Chinese setting. The ICER for intravenous alteplase was 20,693 CNY per QALY over a lifetime horizon, which remained below the WTP threshold (95,749 CNY per QALY). The stability of the findings across all sensitivity analyses affirms the value of IVT with alteplase as a clinically effective and economically sustainable treatment strategy for patients with ischemic stroke.

The cost-effectiveness of alteplase in this context is primarily driven by improved long-term functional outcomes, which translate into substantial reductions in disability-related costs over a patient’s lifetime. This economic benefit is mechanistically explained by our model structure: the superior 3-month mRS distribution established by alteplase directly leads to a greater proportion of patients entering long-term health states associated with lower annual costs and higher quality of life. Although the drug acquisition and monitoring for symptomatic intracranial hemorrhage contribute to higher initial costs, these are effectively offset by the significant increase in the proportion of patients achieving functional independence (mRS 0–2). As more patients return to society with less dependency on long-term rehabilitation and nursing care, considerable indirect cost savings are realized. Thus, short-term investments in thrombolytic therapy yield long-term benefits through preserved medical resources and sustained social productivity.

Our results are consistent with previous economic evaluations of IVT with alteplase across earlier and more restricted time windows (0–3, 3–4.5, 0–6, and 4.5–9 h) (Chen et al., 2023; Joo et al., 2017; Jung et al., 2010), which have almost universally concluded that alteplase is cost-effective. Moreover, our results align with a recent economic analysis of tenecteplase in anterior circulation stroke within a 4.5–24-h window (Chen et al., 2025). Although this prior analysis differs from ours in both the target vascular territory (anterior vs. posterior circulation) and the specific thrombolytic agent assessed, the convergent conclusion is highly instructive. Together, these studies reinforce the health economic rationale for extending the IVT time window based on tissue viability rather than a rigid chronological threshold. They collectively affirm that tissue window-guided extended thrombolysis represents a high-value investment for the Chinese healthcare system.

This evidence is particularly relevant given the distinct clinical and imaging challenges in PCIS. Atypical symptoms, such as dizziness, vertigo, and gait ataxia, frequently lead to delayed diagnosis and hospital presentation beyond the conventional time window (Searls et al., 2012). Furthermore, while advanced perfusion imaging has become a prerequisite for late-window thrombolysis in anterior circulation stroke, its application to the posterior circulation remains technically complex and lacks robust validation (Yan et al., 2025). The lower risk of intracranial hemorrhage in posterior strokes than anterior strokes further supports extending the thrombolysis window even in the absence of advanced perfusion imaging (Lee et al., 2020). Therefore, our findings on cost-effectiveness, when integrated with the clinical efficacy established by the EXPECTS trial (Yan et al., 2025), provide a practical and accessible treatment pathway for settings where perfusion imaging or thrombectomy is unavailable.

Although endovascular thrombectomy has shown efficacy in posterior circulation large-vessel occlusion (Tao et al., 2022; Jovin et al., 2022), its implementation is limited by technical requirements, resource constraints, and cost, particularly in low- and middle-income countries. In such contexts, IVT becomes a critical alternative. The EXPECTS trial confirmed that alteplase improves functional outcomes in PCIS patients without planned thrombectomy (Yan et al., 2025). Our economic evaluation now confirms that this benefit is obtained at a reasonable cost, further supporting the role of extended-window IVT as a foundational strategy in stroke systems of care.

This study has several limitations. As a model-based economic evaluation, the results rely on simulated inputs and structural assumptions, which may limit their direct generalizability to real-world practice. Although clinical effectiveness, utility, and cost parameters were primarily derived from Chinese data to ensure internal consistency with the EXPECTS trial, the findings may not be directly applicable to other healthcare systems. The standard therapy comparator was defined according to the 2018 Chinese stroke management guidelines (Neurology and Society, 2018), which were contemporaneous with the EXPECTS trial (Yan et al., 2025); although guidelines have since been updated (Ch inese Stroke Association, 2024), the comparative assessment remains valid because the analysis focuses on the incremental benefits of alteplase over standard care. In addition, while tenecteplase and other thrombolytics have demonstrated non-inferiority in selected settings, their cost-effectiveness for posterior circulation ischemic stroke within the 4.5–24-h window remains uncertain and warrants further study. Moreover, the EXPECTS trial did not report mRS distributions stratified by onset-to-treatment intervals within 4.5–24 h, precluding time-specific cost-effectiveness analyses; however, consistent treatment effects across time windows support the robustness of the findings. Finally, due to the lack of mRS-specific fatality rates after recurrent stroke, a uniform mortality risk was assumed across health states, with this uncertainty addressed through sensitivity analyses; future studies providing functional-status–specific recurrence and mortality data would improve model precision.

## Conclusion

In summary, for patients with PCIS presenting 4.5–24 h after symptom onset, intravenous alteplase without perfusion imaging guidance is highly cost-effective in China when compared with standard medical therapy alone.

## Data Availability

The original contributions presented in the study are included in the article/Supplementary Material, further inquiries can be directed to the corresponding author.
