# Supplementary material for: Economic value of extended-window intravenous alteplase in posterior circulation stroke: a Markov analysis based on the EXPECTS trial
Source: Front Pharmacol. 2026 Jan 8;16:1734165. doi: 10.3389/fphar.2025.1734165 (PMC12823860; doi:10.3389/fphar.2025.1734165)
Supplement: Supplementary file 1 [file Table1.docx]

sTable 1. Other clinical and cost input parameters and data sources

| Parameters | Value | Range | Distribution | Source |
| --- | --- | --- | --- | --- |
| Outcomes at month 3 for patients in standard treatment | | | | |
| mRS 0 | 0.2564 | 0.1773-0.3355 | / | Ref 10 |
| mRS 1 | 0.3504 | 0.2640-0.4369 |  |  |
| mRS 2 | 0.1197 | 0.0609-0.1785 |  |  |
| mRS 3 | 0.1197 | 0.0609-0.1785 |  |  |
| mRS 4 | 0.0342 | 0.0013-0.0671 |  |  |
| mRS 5 | 0.0342 | 0.0013-0.0671 |  |  |
| mRS 6 | 0.0855 | 0.0348-0.1361 |  |  |
| Outcomes at month 3 for patients in alteplase | | | | |
| mRS 0 | 0.3913 | 0.3021-0.4805 | / | Ref 10 |
| mRS 1 | 0.3478 | 0.2608-0.4349 |  |  |
| mRS 2 | 0.1565 | 0.0901-0.2229 |  |  |
| mRS 3 | 0.0261 | 0-0.0552 |  |  |
| mRS 4 | 0.0174 | 0-0.0413 |  |  |
| mRS 5 | 0.0087 | 0-0.0257 |  |  |
| mRS 6 | 0.0522 | 0.0115-0.0928 |  |  |
| sICH incidence in standard treatment | 0.0087 | 0-0.0409 | β | Ref 10 |
| sICH incidence in alteplase | 0.0172 | 0-0.0257 | β | Ref 10 |
| Death hazard ratios | | | | |
| mRS 0 | 1 | 1–1.2 | / | Ref 18 |
| mRS 1 | 1 | 1–1.2 |  |  |
| mRS 2 | 1.11 | 0.89–1.3 |  |  |
| mRS 3 | 1.27 | 1.02–1.52 |  |  |
| mRS 4 | 1.71 | 1.37–2.05 |  |  |
| mRS 5 | 2.37 | 1.90–2.84 |  |  |
| Annual background mortality | | | | |
| 60- | 0.00760 |  | / | Ref 17 |
| 65- | 0.01266 |  |  |  |
| 70- | 0.02159 |  |  |  |
| 75- | 0.03731 |  |  |  |
| 80- | 0.06340 |  |  |  |
| 85- | 0.15120 |  |  |  |

mRS, modified Rankin Scale; sICH, symptomatic intracranial hemorrhage
